# In Tandem Intragenic Duplication of Doublesex and Mab-3-Related Transcription Factor 1 (*DMRT1*) in an *SRY*-Negative Boy with a 46,XX Disorder of Sex Development

**DOI:** 10.3390/genes14112067

**Published:** 2023-11-12

**Authors:** Veronica Bertini, Fulvia Baldinotti, Pietro Parma, Nina Tyutyusheva, Margherita Sepich, Giulia Bertolucci, Camillo Rosano, Maria Adelaide Caligo, Diego Peroni, Angelo Valetto, Silvano Bertelloni

**Affiliations:** 1 Section of Cytogenetics, Department of Laboratory Medicine, Azienda Ospedaliero Universitaria Pisana, 56126 Pisa, Italy; v.bertini@ao-pisa.toscana.it; 2 Section of Molecular Genetics, Department of Laboratory Medicine, Azienda Ospedaliero Universitaria Pisana, 56126 Pisa, Italy; f.baldinotti@ao-pisa.toscana.it (F.B.); ma.caligo@ao-pisa.toscana.it (M.A.C.); 3 Department of Agricultural and Environmental Sciences, University of Milan, 20133 Milano, Italy; pietro.parma@unimi.it; 4 Division of Pediatrics, Department of Obstretics, Gynecology and Pediatrics, Azienda Ospedaliero Universitaria Pisana, 56126 Pisa, Italy; nina.tyutyusheva@ao-pisa.toscana.it (N.T.); margherita.sepich@gmail.com (M.S.); giuliabertolucci91@gmail.com (G.B.); d.peroni@unipi.it (D.P.); s.bertelloni@ao-pisa.toscana.it (S.B.); 5 Proteomics and Mass Spectrometry Unit, Policlinico San Martino, 16132 Genova, Italy; camillo.rosano@gmail.com

**Keywords:** 46,XX DSD, *SRY*, *DMRT1*, duplication

## Abstract

Disorders of sexual development (DSDs) encompass a group of congenital conditions associated with atypical development of internal and external genital structures. Among those with DSDs are 46,XX males, whose condition mainly arises due to the translocation of *SRY* onto an X chromosome or an autosome. In the few *SRY*-negative 46,XX males, overexpression of other pro-testis genes or failure of pro-ovarian/anti-testis genes may be involved, even if a non-negligible number of cases remain unexplained. A three-year-old boy with an *SRY*-negative 46,XX karyotype showed a normal male phenotype and normal prepubertal values for testicular hormones. A heterozygous *de novo* in tandem duplication of 50,221 bp, which encompassed exons 2 and 3 of the Doublesex and Mab-3-related transcription factor 1 (*DMRT1*) gene, was detected using MPLA, CGH-array analysis, and Sanger sequencing. Both breakpoints were in the intronic regions, and this duplication did not stop or shift the coding frame. Additional pathogenic or uncertain variants were not found in a known pro-testis/anti-ovary gene cascade using a custom NGS panel and whole genome sequencing. The duplication may have allowed *DMRT1* to escape the transcriptional repression that normally occurs in 46,XX fetal gonads and thus permitted the testicular determination cascade to switch on. So far, no case of *SRY*-negative 46,XX DSD with alterations in *DMRT1* has been described.

## 1. Introduction

Disorders of sex development (DSDs) result from a disruption of the delicate balance between mutually antagonistic male and female regulatory networks [1,2,3].

Males with the 46,XX karyotype are uncommon among individuals with DSDs, accounting for roughly 1 in every 20,000 male births. Translocation of the *SRY* gene onto one of the X chromosomes or onto an autosome occurs in about 90% of subjects with 46,XX karyotypes [4]. In recent years, an increasing number of genetic variants associated with *SRY*-negative 46,XX testicular/ovotesticular DSDs have been described, including variants in which pro-testis genes (such as *SOX9*) gain function and variants in which members of the pro-ovarian pathway (such as the *WNT4/RSPO1* signaling pathway) lose function [5]. Moreover, the genetic causes of the majority of these *SRY*-negative cases are unknown.

Doublesex and Mab-3-related transcription factor 1 (*DMRT1*) is a gene involved in testis differentiation and maintenance of the male somatic cell fate after testis differentiation [6,7]. In humans, this gene gives rise to three alternative transcripts (https://www.ensembl.org, accessed on 25 July 2023), but only ENST00000382276.8 encodes a protein with a DM domain. The DM domain is a zinc finger-like DNA-binding motif characterized by a bipartite motif that consists of an intertwined double zinc-binding module followed by an α helix, and was first identified in the sex regulator doublesex (*Dsx*) of *Drosophila* and male abnormal-3 (*MAB-3*) of *C. elegans* [7]. Genes encoding a DM domain are transcription factors that are conserved in a broad range of metazoans, and they act as triggers for sex determination in multiple phyla with a gonad-specific and sexually dimorphic expression pattern [6,8,9,10]. 

In mammals, *SRY* is the master gene that switches on male sex determination, and *DMRT1* is thought to be less relevant to this process [5]. However, experimental data in a transgenic mouse model demonstrated that ectopic overexpression of *Dmrt1* in XX fetal gonads determined testicular differentiation in the embryo and male secondary sex development after birth [5,11]. Studies in mice indicate that *Dmrt1* is also involved in maintaining testis differentiation long after its determination, showing that mammalian gonads may have some degree of plasticity throughout life [6,12,13,14].

In humans, heterozygous deletions in chromosome 9p24.3 involving *DMRT1* are reported in 46,XY patients with gonadal dysgenesis or ovotestes, suggesting that *DMRT1* haploinsufficiency is associated with defective testicular development and male-to-female DSDs [6]. This role is confirmed by *DMRT1* missense point mutations in females with a 46,XY karyotype and gonadal dysgenesis [6]. An *SRY*-negative 46,XX DSD due to an alteration of *DMRT1* has not been described in humans.

Here, the case of a boy with an *SRY*-negative 46,XX testicular/ovotesticular DSD carrying a *de novo*, in tandem, intragenic duplication of *DMRT1* is reported.

## 2. Case Report and Materials and Methods

### 2.1. Patient

The subject was referred to us at the age of 3 years and 3 months. A prenatal ultrasound highlighted normal male genitalia, but cytogenetic analyses, which were performed for advanced maternal age, revealed a 46,XX karyotype. A chromosome analysis was performed on cultured amniocytes using conventional techniques. Q-banding at the 320 band level showed a 46,XX karyotype in all 15 clones analyzed. SRY was not detected using either FISH or PCR analysis.

The 46,XX subject exhibited a normal male phenotype. Both testes were located in the scrotum and had a low volume for a prepubertal age (mean testicular volume, SDS: −1,1) [15]. Ultrasound investigations showed a normal testicular structure and the absence of Müllerian derivatives. The endocrine evaluation demonstrated normal values for gonadotropin and androgens for a pre-pubertal male, as well as normal levels of antimüllerian hormone (AMH; 756.8 pmol/L; reference range 321–1218 pmol/L) and inhibin B (108.0 pg/mL; reference range 23–252 pg/mL) [16]. Written informed consent was obtained from the patient’s parents before clinical and genetic investigations.

### 2.2. Next-Generation Sequencing (NGS)

The patient’s DNA, which was used in all the analyses, was isolated from peripheral blood.

A 34-gene custom panel for DSDs, including only the coding sequence and the donor and acceptor splice sites (Table 1), was performed using SureSelect XT target enrichment (Agilent Technologies, Santa Clara, CA, USA) according to the manufacturer’s instructions. The products were sequenced using the MiSeq Illumina platform (Illumina, San Diego, CA, United States). Annotation and filtering of variants were performed with the Variant Interpreter platform. Next-generation sequencing (NGS) coverage was analyzed in detail using Integrative Genome Viewer version 2.3.

### 2.3. Whole Genome Sequencing (WGS)

The library construction workflow was performed according to the standard protocol provided by Illumina (San Diego, CA, USA), including sample quality control, library construction, library quality control and library sequencing.

Detection of SNPs (single nucleotide polymorphisms) and small InDels (small insertions and deletions) was mainly conducted using the GATK software package v.4.3.0.0 [17]. According to the mapping result from Clean Reads to the reference genome, Samtools (v1.9) [18] was used to filter abundant reads and ensure the accuracy of the test result. The HaplotypeCaller (local haplotype assembly) algorithm from GATK was applied to detect SNP and InDel variations.

Genomic structural variations (SV), such as insertions (INS), deletions (DEL), inversions (INV), and chromosomal translocations (TRA), were detected using Manta [19].

The probable impact of the variations found and the possible involvement of genetic factors was verified with VEP release 110 [20].

### 2.4. Multiplex Ligation-Dependent Probe Amplification (MLPA)

Multiplex ligation-dependent probe amplification (MLPA) was performed using a SALSA MLPA P334-A3 Gonadal kit, which included probes for the *CYP17A1*, *DMRT1*, *HSD17B3*, and *SRD5A2* genes, and a SALSA MLPA P185-C2 Intersex kit, which contained a probe mix for the following genes: *NR0B1*, *CXorf21*, *SOX9*, *SRY* and *ZFY* (Yp11.3), *WNT4*, and *NR5A1* (MRC-Holland, Amsterdam, The Netherlands). Amplified products were separated by size using the capillary electrophoresis system with an ABI-3500 Genetic Analyzer (Applera, Milan, Italy), and data were analyzed using the software Coffalyser.Net v. 220513.1739 (MRC-Holland, Amsterdam, The Netherlands). A peak was considered abnormal when the ratio was <0.65 (deletion, or a copy number change from two alleles to one) or >1.30 (duplication, or a copy number change from two to three or more alleles) compared to the peaks of the reference probes.

### 2.5. Comparative Genomic Hybridization Array (CGH-Array)

DNA from a healthy 46,XX subject was used as the control (Agilent Technologies, Santa Clara, CA, USA). Samples of 500 ng of genomic DNA were gathered from the patient (test sample) and the control (reference sample). Samples were differentially labeled with Cy5-dCTP or Cy3-dCTP using random primer labeling according to the manufacturer’s protocol (Agilent, Santa Clara, CA, USA). Labeling reactions were applied to the oligo arrays and incubated in an oven for 24 h at 67 °C. The slide was washed, scanned using an Agilent scanner, and analyzed using Agilent’s dedicated software (Feature Extraction, v. 4.0.3.12, Agilent, Santa Clara, CA, USA). The comparative genomic hybridization (CGH) array was performed on a customized 180K SurePrint G3 Human CGH Micro-array (Agilent, Santa Clara, CA, USA), which was enriched in the genes correlated to DSDs (Table 1). For these genes, the overall median probe spacing was about 1.8 kb both in the intronic and the exonic regions. The CNVs were identified with Cytogenomics 3.0.6.6 (Agilent, Santa Clara, CA, USA) using the aberration detection method 2 (ADM-2) algorithm. CNV classification was performed according to the guidelines of the Italian Society of Human Genetics (https://www.sigu.net, accessed on 25 July 2023). The results were reported according to GRCh37/hg19.

### 2.6. Amplification and Sequencing of the Junction Fragment

PCR was performed using 50 ng of DNA, 0.2 mM of each primer (*DMRT1*-forward: CAACACCTCCAAGCCCTCTT; *DMRT1*-reverse: CACCACAAGAGTGGTACACAGA), and 5x HOT FIREPol Blend Master Mix (Solis BioDyne—Life Science Reagents) in a 50 μL volume mixture. The purified PCR product was bidirectionally sequenced (BigDye Terminator v3.1 Cycle Sequencing Kit; Life Technologies, Milan, Italy) and analyzed using a 3500 Genetic Analyzer (Applera, Milan, Italy). The direct and inverse sequences were analyzed and compared with the DMRT1 reference sequence (NG_009221.1).

## 3. Results

No pathogenic or uncertain variants were identified through using a custom NGS panel for DSDs (Table 1). 

WGS was performed and sequencing produced a total of 641,993,752 clean reads for a total of 96,036,225,225 bases. In total, 90.07% of these reads were mapped correctly, providing 10× coverage for 92.81% of the genome. These data were used to confirm what was observed in the analysis of the NGS panel and to exclude alterations in promoters and introns of the known DSD genes, as well as regulatory regions, including the SOX9 upstream regulatory region.

**Figure 1:**
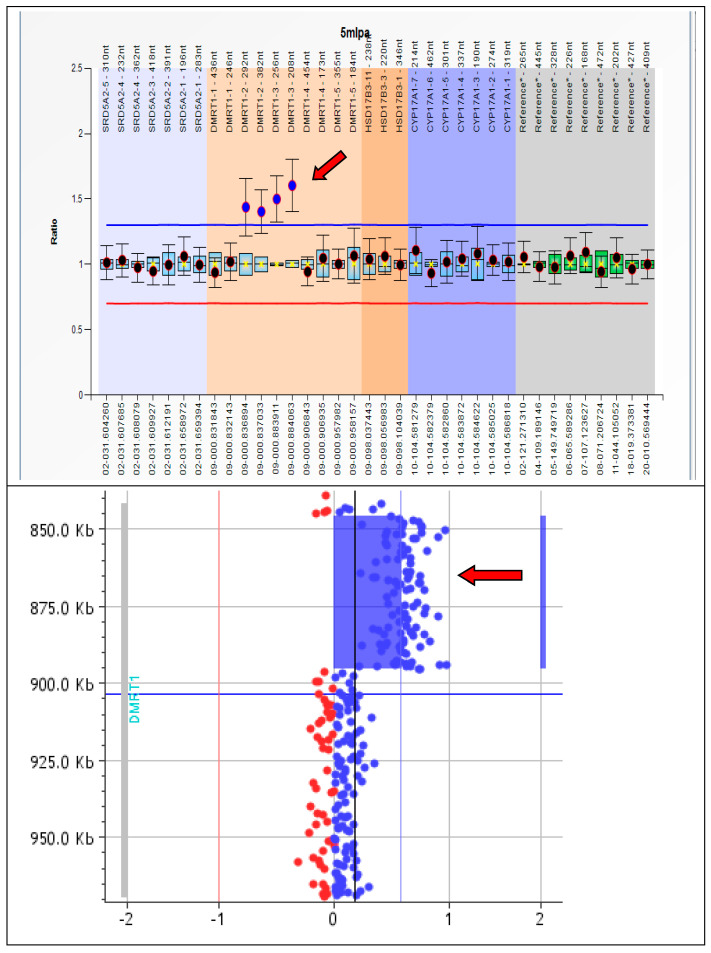
Results from the MLPA test (**top**) and CGH-array (**bottom**), showing the intragenic duplication of *DMRT1* (arrows).

The MLPA analysis with a SALSA MLPA P185-C2 Intersex kit confirmed the absence of *SRY* and showed normal copy numbers for NR0B1, CXorf21, SOX9, WNT4, and NR5A1. The MLPA analysis with a SALSA MLPA P334-A3 Gonadal kit resulted in a ratio value for the probe hybridizing to *DMRT1* exon 2 (13074-L14293 and 13078-L14297 probes) and exon 3 (13072-L14291 and 13069-L14288 probes), consistent with a heterozygous duplication. The probes targeting the other exons of the *DMRT1* showed a normal copy number (Figure 1).

**Figure 2:**
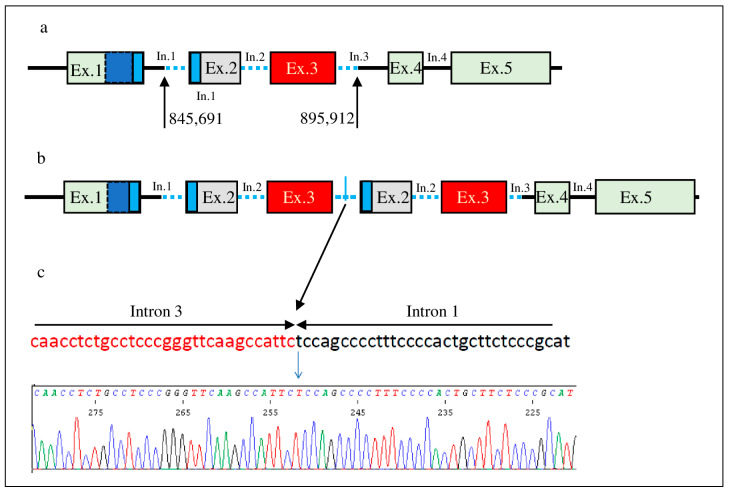
(**a**). Schematic representation of the normal allele of the *DMRT1* gene with the localization of the breakpoints identified (introns are not in scale). (**b**). Schematic representation of the mutated allele of *DMRT1*. (**c**). Sequencing results from the PCR amplification of the junction of intron 1 and intron 3. Blue and cyan rectangles show the intertwined double zinc-binding module and the α helix, respectively.

A custom CGH-array analysis confirmed an intragenic *DMRT1* duplication of about 50 Kb. The proximal breakpoint is located between position 845,172 bp (oligo not duplicated) and position 845,893 bp (oligo duplicated). The distal breakpoint is between position 895,518 bp (oligo duplicated) and position 896,154 bp (oligo not duplicated). The CGH-array result was arr[GRCh37] 9p24.3(845,893_895,518)x3. The two breakpoints were mapped to the intronic regions (Figure 1 and Figure 2).

Amplification of a genomic fragment using a primer distal to exon 3 (forward) and one proximal to exon 2 (reverse) indicated that the duplication occurred in tandem. Sanger sequencing of the amplificated fragment determined the accurate mapping of the junction between intron 3 and intron 1 and, consequently, the exact position of the duplication: 845,691–895,912 (50,221 bp) (Figure 2).

Exon 2 of *DMRT1* (ENST00000382276.8) starts with the nucleotide triplet GTG, which encodes Val-119, and exon 3 ends with the nucleotide triplet CAG, which encodes Gln-274. Thus, the first amino acid residue of exon 2 and the last one of exon 3 are encoded by nucleotide triplets that are not shared by the adjacent exons. 

Therefore, the reported duplication does not stop or shift the coding frame, but it may determine a longer transcript in which exons 2 and 3 are duplicated in tandem (Figure 2). This transcript could be translated into a protein with 156 additional amino acids that includes the portion of the DM domain encoded by exon 2. The parental analysis, which was performed with MLPA, showed the *de novo* origin of this duplication (Figure S1, Supplementary Materials). 

## 4. Discussion

*DMRT1* is an ancient sexual regulator that plays a role in sex determination [2,6]. In humans, *DMRT1* is a pro-testis gene; its haploinsufficiency in 46,XY individuals deranges normal male gonadogenesis. However, its role as a master gene triggering testis differentiation in *SRY*-negative 46,XX males has not yet been reported [6].

Here, an *SRY*-negative subject with a 46,XX testicular/ovotesticular DSD and a normal male phenotype of both internal and external genitalia is described. Although no histological gonadal data are available, it can be inferred that the prepubertal testicular tissue is “functional” due to the normal male values for AMH and inhibin B, which indicate the persistence of postnatal Sertoli cell function. These findings exclude the possibility of streak gonads, which are biochemically characterized by abnormally low levels of both Sertolian hormones. The normal masculinization of genitalia and the absence of Müllerian derivatives indicate that optimal levels and functions of fetal AMH and androgens were present during the prenatal period of sex differentiation [1,2,5].

This subject shows a *de novo*, in tandem, intragenic duplication of *DMRT1* (Figure 1 and Figure 2). Extensive molecular investigations of genes related to sex determination and differentiation did not reveal other pathogenetic variants.

Experimental data have shown the ability of *Dmrt1* to trigger testis differentiation in *SRY*-negative 46,XX mice. Ectopic overexpression of *Dmrt1* in XX fetal gonads permitted testicular differentiation and postnatal maintenance of male secondary sex characteristics [11]. In goats, a gene expression analysis that compared XX wild type gonads to *FOXL2* gonads with a loss of function (XX PIS−/−) revealed the up-regulation of *DMRT1* before *SOX9* expression. This result suggests that DMRT1 may be able to promote *SOX9* up-regulation in mammals independently from *SRY* [21].

In humans, *DMRT1* presents three alternative transcripts, but only ENST00000382276.8 produces a protein with a DM domain (Ensembl, https://www.ensembl.org, accessed on 25 July 2023). This domain is characterized by a bipartite motif that consists of an intertwined double zinc-binding module followed by an α helix. The zinc-binding module corresponds to amino acids Ser70–Cys105, which are encoded by exon 1, whereas the α helix corresponds to amino acids Asn106–Leu131, which are encoded by exons 1 and 2. 

The functional effects of this duplication on *DMRT1* activity are difficult to predict. Intragenic duplications often cause a loss of function. However, *DMRT1* loss of function does not switch on male sex determination. Notably, 46,XX fetuses and adults with distal 9p deletions show a female phenotype and normal ovaries. In prior studies, three subjects (two with deletions and one with a point mutation variant) inherited *DMRT1* alterations from their fertile mothers [22,23,24]. Thus, *DMRT1* loss of function in a 46,XX context can be compatible with normal ovarian differentiation and female fertility. This result has been confirmed in mouse models [8], but not in a rabbit model, in which XX *DMRT1*−/− gonads did not undergo meiosis [25].

Since *DMRT1* is a pro-testis gene, the presence of “functional” testes indicates that *DMRT1* gained function. Duplication of exons 2 and 3 does not stop or shift the coding frame and could result in a longer transcript.

In 46,XX subjects, *DMRT1* is transcriptionally repressed and it could be hypothesized that, in the present case, this different transcript escapes the normal repression mechanism present in 46,XX developing gonads. This theory needs to be explored further, most likely with an animal model, albeit the variable behavior of *DMRT1* in different animal models may complicate this activity.

**Figure 3:**
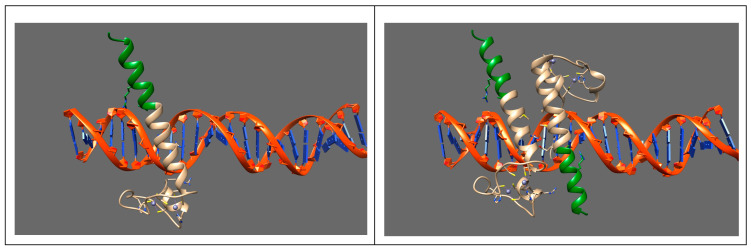
Three-dimensional structure of the DM domain (fragment: Ser70-Leu131). (**Left panel**): A single helix (ribbons) binds the major groove of the DNA molecule, the interacting amino acids are drawn as sticks, and zinc ions are reported as cyan spheres. (**Right panel**): Two adjacent helices are binding the major groove of the same 25-mer, as determined using X-ray crystallography. The amino acid residues encoded by exon 1 are depicted in beige and those encoded by exon 2 are in green.

This transcript could be translated into a protein with 156 additional amino acids that includes the portion of α helix encoded by exon 2. The interaction between the DMRT1 DM domain and DNA in mouse and humans has been analyzed using X-ray crystallography (PDB code: 4YJ0) (Figure 3) [26]. According to this model, the α helix inserts into the DNA major groove, conferring sequence-specific DNA, whereas the zinc-binding module spans the minor groove, primarily through phosphate backbone contacts [26]. A noteworthy feature of DMRT1–DNA binding is that two adjacent α helices of two DMRT1 proteins lie anti-parallel together in the major groove of the consensus element (Figure 3). No other protein interacts with DNA through a pair of α helices so closely that they are both inserted into the same section of a major groove [6]. The mutated protein, with the additional partial α helix encoded by exon 2, could cause a gain of function, mimicking the effect of a DMRT1 dimer.

## 5. Conclusions

This is the first report of an *SRY*-negative subject with a 46,XX testicular/ovotesticular DSD carrying a *de novo* in tandem, intragenic duplication of *DMRT1*. Even if these data do not prove conclusively that this duplication causes the phenotype, this case may represent an important starting point for future investigations.

## Figures and Tables

**Table 1:** Custom NGS panel and CGH-array for DSDs. Top: List of genes sequenced; bottom: list of genes with an overall median probe spacing of about 1.8 kb.

**Custom NGS panel for DSDs** (GRCh37/hg19)
AKR1C2 (NM_001354.4), AMH (NM_000479), AMHR2 (NM_020547.2), AR (NM_000044.2), ARX (NM_139058.2), ATRX (NM_000489.3), BMP15 (NM_005448.2), CBX2 (NM_005189.2), CYB5A (NM_001914.3), CYP11A1 (NM_000781.2), CYP11B1 (NM_000497.3), CYP17A1 (NM_000102), CYP19A1 (NM_031226.2), DHH (NM_021044.2), DHX37 (NM_032656.3), DMRT1 (NM_021951.2), GATA4 (NM_002052.3), HSD17B3 (NM_000197), HSD3B2 (NM_000198.3), LHCGR (NM_000233.3), MAMLD1 (NM_005491.3), MAP3K1 (NM_005921.1), NR0B1 (NM_000475), NR2F2(NM_021005), NR5A1 (NM_004959), POR (NM_000941.2), PPP1R12A (NM_002480.3), RSPO1 (NM_001038633.3), SOX9 (NM_000346), SRD5A2 (NM_000348), SRY (NM_003140), STAR (NM_000349), WNT4 (NM_030761.4), WT1 (NM_024426.4), ZFPM2 (NM_012082.3).
**Custom CGH-array for DSDs** (GRCh37/hg19)
AKR1C2, AKR1C4, AMH, AMHR2, AR, BMP15, CBX2, CTNNB1 β-catenin, CYP11A1, CYP11B1, CYP17A1, CY19A1, CYP21A2, DHH, DMRT1, DMRT2, GATA4, FGF9, FGFR2, FOXL2, HHAT, HSD17B3, HSD3B2, LHCGR, MAMLD1, MAP3K1, NR0B1 (DAX1), NR5A1 (SF1), PGD2, POR, RSPO1, SOX3, SOX9, SOX10, SRD5A2, SRY, STAR, WNT4, WT1, WWOX, ZFPM2 (FOG2).

## Data Availability

All data generated or analyzed during this study are included in this article. Further enquiries can be directed to the corresponding author.

## Supplementary Materials

The following supporting information can be downloaded at https://www.mdpi.com/article/10.3390/genes14112067/s1: Figure S1: Results from the MLPA test of the proband’s parents.
