# Supplementary figures and images for: In Tandem Intragenic Duplication of Doublesex and Mab-3-Related Transcription Factor 1 (DMRT1) in an SRY-Negative Boy with a 46,XX Disorder of Sex Development
Source: Genes (Basel). 2023 Nov 12;14(11):2067. doi: 10.3390/genes14112067 (PMC10671459; doi:10.3390/genes14112067)

Figure S1: The MLPA result for the parents. a: Mother, b: Father.

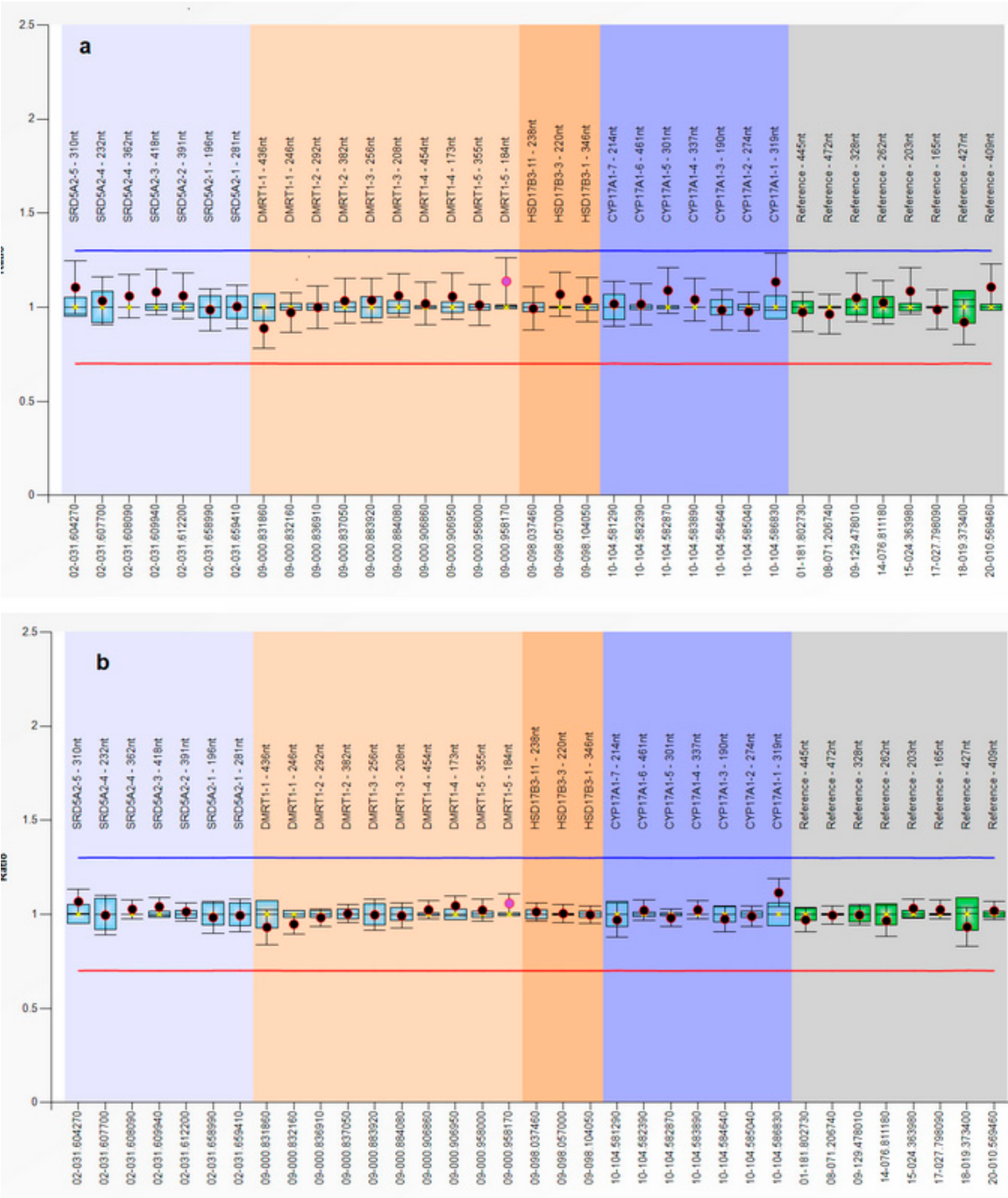

Supplement: Supplementary file 1 [file genes-14-02067-s001.zip › genes-2554789-supplementary.pdf]
